# Temporal analysis of the incidence of meningitis in the Tehran metropolitan area, 1999-2005

**DOI:** 10.1186/1478-7954-7-19

**Published:** 2009-12-23

**Authors:** Alireza Mosavi-Jarrahi, Abdolreza Esteghamati, Freshteh Asgari, Mohammadali Heidarnia, Yasamin Mousavi-Jarrahi, Mohammadmehdi Goya

**Affiliations:** 1 Department of Epidemiology, School of Public Health, Shaheed Beheshti University of Medical Sciences and Health Services, Tehran, I. R. of Iran; 2 The Cancer Research Center of the Cancer Institute, the Imam Khomeini Medical Center, Tehran University of Medical Sciences and Health Services, Tehran, Iran; 3 Vaccine Preventable Diseases, Center for Disease Control, Ministry of Health, Tehran, Iran; 4 Department of Social Medicine, Medical School, Shaheed Beheshti University of Medical Sciences and Health Services, Tehran, Iran

## Abstract

**Objectives:**

The aim of this study was to describe the temporal determinants of meningitis incidence in the population living in the Tehran metropolis.

**Methods:**

All cases of meningitis reported to health districts throughout the Tehran metropolis from 1999 to 2005 were abstracted from patient files. Referral cases (patients who did not reside in the Tehran metropolis) were excluded. For each year, sex- and age-specific incidences were estimated. Temporality and its determinants were analyzed using Poisson regression.

**Results:**

Age-specific incidence is highest among males younger than 5 years of age at 10.2 cases per 100,000 population per year. The lowest incidence was among females aged 30 to 40 years at 0.72 cases per 100,000 population per year, with an overall male-to-female incidence ratio of 2.1. The temporal analysis showed seasonality, with a higher risk of meningitis in spring at a rate ratio of 1.31 with a 95% confidence interval (CI) of 1.20 to 1.41 and in autumn (rate ratio = 1.16, 95% CI 1.06, 1.27). For periodicity, we found a peak of occurrence around the years 2000 and 2003.

**Conclusion:**

The epidemiology of meningitis in Iran follows similar patterns of age, sex, and seasonality distribution as found in other countries and populations.

## Introduction

Meningitis is an important cause of morbidity and mortality in developing countries, with an estimated case fatality rate of 4% to 27%[1]. Outbreaks of meningitis cause high degrees of anxiety in local populations, though a large portion of cases are sporadic. Although different infectious agents can cause meningitis, most meningitis cases are caused by meningococcal meningitis and Haemophilus influenzae type b (Hib), especially in children under 15 years. No information has been published regarding the incidence of meningitis in Iran. The estimated incidence for the country is based on mathematical modeling or reports of limited cases[2]. The incidence of meningitis worldwide varies depending on the location of a given community. High incidence of meningococcal meningitis -- as high as 100 cases per 100,000 population -- is reported in the sub-Saharan meningitis belt of Africa, which includes several countries of middle Africa from south of Mauritania to south of Sudan. In contrast, low incidences are reported in Europe (as low as 1.2 per 100,000 population). While information regarding meningitis epidemics or annual incidence is not well-documented in the countries of the Middle East and North Africa, the World Health Organization (WHO) reports that a meningitis epidemic is widespread in Egypt, Morocco, and Sudan as well as in Eastern Mediterranean countries, such as Saudi Arabia and Yemen[1]. In an outbreak of meningitis following the return of Hajj pilgrims in August 1987, many countries in the region faced an unusual spread of meningococcal infection, resulting in mandatory vaccination of pilgrims going to Hajj[3,4].

It is not clear how the bacteria spread through populations in time and space or which determinants are most important in different areas and populations. Suggested environmental risk factors for meningitis include social deprivation, overcrowding[5], passive smoking[6], and weather conditions[7]. While the distribution of meningitis is known, the determinants for epidemic meningitis are not established all over the world. The broad worldwide picture and the high-risk area of the African meningitis belt indicate seasonality as well as a periodic wave of changes in incidence every five to 12 years[8,9] as key determinants. Epidemic meningitis has been associated with seasons of dry conditions and low temperatures[10]. It has been postulated that, in the sub-Saharan region of Africa, dry seasons are correlated with winds and the spread of dust as well as the congregation of people in small groups, increasing likelihood of person-to-person transmission.

The aim of this study was to determine the incidence of meningitis and to describe the temporal determinants of meningitis in the greater Tehran metropolitan area from 1999 to 2005.

## Methods

### Subjects and case definitions

All cases of meningitis reported to District Health Centers (DHC) throughout the Tehran metropolis were included in this study. In Iran, reporting of meningitis cases to public health authorities is mandatory. All hospitals and private clinics must report any cases of meningitis to their corresponding DHC, where a Standard Case Investigation Procedure (SCIP) is performed. The SCIP includes recording of the patient's demography, diagnosis, and clinical history as well as contact investigation. The information generated in the SCIP is kept in the DHC, with additional tabulated reports sent to the Office of Disease Control at the Ministry of Health. Reported cases are mainly defined based on WHO definitions as suspect, probable, and confirmed[11]. A detailed description of the adapted case definition is presented in Table 1. Most cases lack a definite microbiological diagnosis due to early and aggressive treatment or other practical shortcomings. For this study, the file of each reported case was identified and reviewed, and the following patient information was extracted: identifier (name and address); demographics (age, sex); date of disease onset (date of hospitalization or reporting of case to the health district); and survival status (alive or dead).

**Table 1:** Standard case definition of meningococcal meningitis based on WHO recommendations** and adapted by the Ministry of Health.

**Suspected case of acute meningitis**:
1) Sudden onset of fever (>38.5°C rectal or 38.0°C axillary)
WITH
2) Stiff neck
In patients under 1 year of age, a suspected case of meningitis occurs when fever is accompanied by a bulging fontanelle

**Probable case of bacterial meningitis**
1) Suspected case of acute meningitis as defined above
WITH
2) turbid CSF

**Probable case of meningococcal meningitis**
1) Suspected case of either acute or bacterial meningitis as defined above
WITH
2) Gram stain showing Gram-negative diplococcus
OR
3) ongoing epidemic
4) petechial or purpural rash

**Confirmed case:**
1) Suspected or probable case as defined above
WITH EITHER
2) positive CSF antigen detection for *N. meningitidis*
OR
3) positive culture of CSF or blood with identification of *N. meningitidis*

** Adapted from the Control of epidemic meningococcal disease, WHO practical guidelines. 2nd edition, World Health Organization, Document number: WHO/EMC/BAC/98.3

A case of meningitis was considered an incidence case if it was reported to one of the 15 DHCs throughout Tehran from 1999 through 2005 with proof of residency (having an address in the greater Tehran metropolitan area).

### The population and study area description

The study population consisted of the residents of the Tehran metropolitan area. The population of Tehran was 6,758,840, 7,659,804, 7,506,608, 7,356,475, 7,209,346, 6,993,066, and 6,783,274 for the years 1999 to 2005, respectively, with more than 20% of the population under 15 years of age. To estimate the incidences, population figures were obtained from the Bureau of Vital Statistics. The bureau provides population estimates for each year based on 10-year census data and the rate of mortality, birth, and immigration.

Geographically, Tehran is located in the foothills of the Alborz Mountain Range and is 1,500 kilometers from the Persian Gulf at the geographic coordinates of 48° (latitude) and 34° (longitude), with an area of 150 square kilometers. The average annual precipitation is about 0.5 centimeters, occurring mainly in the winter and spring with a semidesert climate for summer and fall[12].

### Analysis

Incidence of meningitis was calculated as seven-year averages for five-year age groups and presented as age- and sex-specific curves. In addition, age was categorized as less than 15 years and more than 15 years, and yearly incidences were calculated for males and females in each age category.

The temporal analysis of meningitis incidence was performed using Poisson regression with the following variables: age (categorized as <15, 15-50, and more than 50 years); sex; seasonality (spring, summer, fall, and winter); and year of occurrence. The computer software STATA version 8 was used to perform Poisson regression, and MS Excel was used to calculate frequency and incidence.

## Results

A total of 4,633 meningitis cases were recorded for the seven-year study period (1996-2005 inclusive). Out of the 4,633 cases, mortality information was available for 2,906 cases. Of these, 131 patients died of meningitis, resulting in a case-fatality rate of 4.5% for Iran.

Out of these 4,633 cases, 1,687 had verified Tehran residences, and the onset of disease was between 1999 and 2005, making them eligible incidence cases. The remaining cases were referral cases from other cities, or the disease onset was not in the time period of the study, and therefore those cases were excluded.

**Figure 1:**
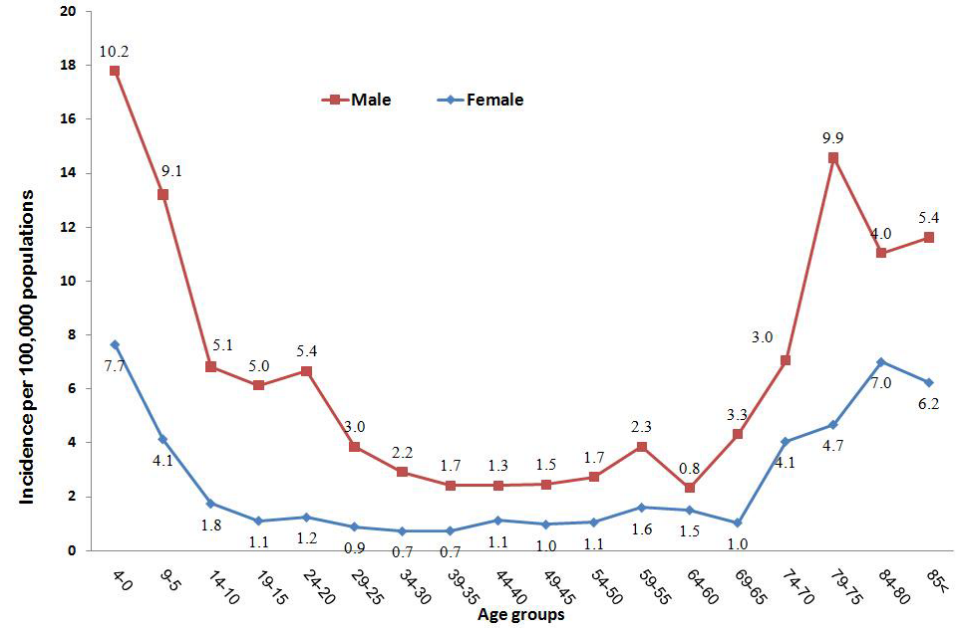
**The age-specific incidence of meningitis based on gender (incidences averaged over seven years)**. The values in the lines present the actual incidence values.

Within the under-15 age group, the year 2000 with a total of 219 cases had the highest disease frequency, with a yearly incidence of 20.1 and 9.9 cases per 100,000 population for males and females, respectively. The year 2004 with a total of 91 cases was the lowest year in terms of disease frequency, with a yearly incidence of 9.2 and 4.5 per 100,000 population for males and females, respectively (Table 2). The age-specific incidence averaged over the study period (1999-2005 inclusive) showed the highest incidence among the male population less than 5 years of age, with average yearly incidence of 10.2 cases per 100,000 population, and the lowest incidence was among women aged 30-40 years, with average yearly incidence of 0.72 cases per 100,000 population. On average, the frequency of male cases was more than that of females, with an overall male-to-female incidence ratio of 2.1. Comparing the age-specific incidence curve between males and females, the age group 15-30 years showed the largest differences between the sexes (Figure 1).

**Table 2:** Number of cases and incidences of meningitis based on age and sex over the study period (1999 to 2005), Tehran metropolis

	**Male**				**Female**			
		
**Variable**	**0-15**		**>50**		**0-15**		**>50**	
**Name**								
	**No. (%)**	Incidence*	**No (%)**	Incidence*	**No. (%)**	Incidence*	**No. (%)**	Incidence*
	
1999	39 (12.5)	10.3	16 (8.0)	1.7	78 (12.3)	5.4	56 (10.4)	0.5
2000	70 (22.4)	20.1	37 (18.6)	3.5	149 (23.4)	9.9	110 (20.4)	1.2
2001	49 (15.7)	18.6	31(15.6)	3.3	135 (21.2)	7.1	104 (19.3)	1.0
2002	39 (12.5)	8.3	40 (21.1)	2.6	59 (9.3)	5.8	78 (14.4)	1.4
2003	36 (11.5)	9.5	27 (13.6)	2.4	66 (10.4)	5.4	72 (13.3)	0.9
2004	29 (9.3)	9.2	22 (11.1)	2.1	62 (9.7)	4.5	61 (11.3)	0.8
2005	50 (16.0)	13.3	26 (13.1)	2.1	87 (13.7)	8.0	59 (10.9)	1.0
All years combined	312 (100)	12.8**	199 (100)	2.5**	636 (100)	6.6**	540 (100)	1.0**

* Incidences are reported per 100,000 population

** Averaged over seven years of the study period

### Temporal analysis

The frequency of meningitis for each year and the year of occurrence were determined and customized for Poisson regression. Several models were evaluated using different combinations of variables and their interaction terms. The final model included all variables without any interaction terms. Based on the final model, males had 2.26 times more risk for meningitis compared to females (95% CI 2.12, 2.41). The young had a higher risk compared to older people, with a rate ratio of 8.48 (95% CI 7.53, 9.54) for <15 years, and a rate ratio of 4.50 (95% CI, 3.97, 5.09) for 15 to 50 years.

As shown in Table 3, we found that seasonal variations in the occurrence of meningitis showed a higher risk in spring, with a rate ratio of 1.31 (95% CI, 1.20, 1.41), and in fall, with a rate ratio of 1.16 (95% CI, 1.06, 1.27).

**Table 3:** Rate ratios and their 95% confidence intervals for factors associated with temporal variations of meningitis.

		95% CI	
Variables	Rate Ratios		
		Lower	Upper
Seasonality			
Spring	1.31	1.20	1.42
Fall	1.16	1.06	1.27
Summer	1.05	0.96	1.15
Winter	1.00		
			
Age group			
< 15	8.48	7.53	9.54
16-50	4.50	3.97	5.09
>50	1.00		
			
Gender			
Male	2.26	2.12	2.41
Female	1.00		
			
Year of incidence			
1999	0.99	0.85	1.15
2000	1.78	1.56	2.04
2001	1.87	1.64	2.13
2002	0.96	0.82	1.12
2003	1.98	1.74	2.26
2004	1.96	1.72	2.23
2005	1.00		

## Discussion

This study determined detailed age- and sex-specific incidences of meningitis in the population of the Tehran metropolis for the first time. The peculiar pattern of age- and sex-specific incidence, presented as age-specific curves, shows a sharp difference in incidence ratios between the sexes according to age. The three- to fourfold increase in male cases aged 15 to 35 years presents a challenging issue in the epidemiology of meningitis in Iran. It is particularly important to note that a large portion of the male population is drafted into military service at approximately 20 years of age, at which point these men receive a compulsory meningitis vaccine. The compulsory vaccination of serotypes A and C was started before 1999. On the other hand, the female population is not drafted into military service and has no required exposure to vaccination. The effective decline in the incidence of meningitis due to vaccination has been documented in populations where vaccination has been applied[13]. There is an overall male predominance for meningitis and other infectious diseases reported from various countries. Nevertheless, with more than 70% of men in the Iranian population vaccinated after age 18, one would expect to see a different sex- and age-specific pattern of meningitis incidence after this age. Exploring the underlying cause of this discrepancy can help better explain the epidemiology of meningitis in the population.

We report a low case-fatality rate for the studied population compared to that of other parts of the world[1]. Most of the countries of Africa and Southeast Asia have case-fatality rates of higher than 8%, according to the WHO[1]. Because the survival status of a large number of cases was not available for this study, the loss of data may have an effect on our estimated case fatality rate.

The temporal variations of meningitis have been associated with seasonal variations as well as periodicity. Seasonal variations in incidence seen in the sub-Saharan region of the meningitis belt include a higher incidence in dry seasons associated with wind and dust in the dry season. This pattern of seasonality has also been observed in other areas of the world, such as the US[10]. Our data showed a higher risk of disease in the spring and fall compared to summer and winter. While there is not much difference in terms of humidity during the four seasons, Tehran has dry weather, with precipitation barely exceeding 0.5 centimeters per year[12]. Furthermore, one of the main sources of air pollution in Tehran is a high concentration of particulate matter consisting mainly of dust[14] that could contribute to higher risk of meningitis in this population. To what extent climatic factors affect the risk of meningitis in this population requires further study; however, there are reports of higher frequency of meningitis in the city of Qom, which is 250 kilometers south of Tehran and very close to the desert with almost zero rain and very dry weather[15].

While the seasonality of meningitis is established, studies of temporal changes in incidence over the years have shown elements of periodicity across longer time periods[16]. The periodicity of epidemic meningitis in countries located in the sub-Saharan meningitis belt has been demonstrated, occurring on average every 10 to 12 years[8]. Periodicity is related to the dynamic interaction of the disease with a population's indicators of susceptibility, herd immunity, and the antigenic changes in the causative agent. The establishment of a temporal trend by looking for an increase or decrease in incidence may not be a suitable trend analysis for infectious diseases compared to that of chronic noninfectious diseases, such as cancer, where herd immunity has no place in the dynamics of temporal changes of incidence. However, our data over the relatively short period of time of seven years showed there are some fluctuations in the incidences with two peaks of high incidence in the years 2000 to 2001 and 2003 to 2004. The high level of completeness in meningitis reporting, combined with our ability to exclude referral cases, results in a good degree of case ascertainment. Therefore, this fluctuation may be part of the broader dynamic of epidemic meningitis in the studied population. Additional studies over a longer time period are needed to demonstrate how epidemic meningitis behaves in terms of temporality.

The results of our study must be interpreted in light of: 1) completeness of case ascertainment, 2) quality of data available, and 3) the overall nature of epidemic meningitis.

The completeness of reporting of meningitis cases has been the subject of several studies in various countries, with some countries reporting 95% completeness. In Iran, it is mandatory to report all meningitis cases to the DHCs. As a standard procedure, the SCIP information is actively collected by a trained epidemiologist and includes the patient's history, microbiology, and clinical as well as contact information. No data are available about the completeness of meningitis reporting in Iran; however, the incidence estimates obtained in our study conform with another study using a different methodology[17]. The fact that our study was able to differentiate eligible cases from referral cases adds to the quality of data available, resulting in a higher validity of the incidence estimates and descriptive measures.

On the other hand, the quality of our data had two limitations: the inherent problem of retrospective data, and symptomatic diagnosis rather than specific causative-agent confirmed diagnosis. Epidemiological investigations of meningitis addressing case definition have reported different rates of case confirmation in compulsory meningitis reporting programs. One large study examining hospitalized cases for 32 years revealed that 54% of cases were considered confirmed as meningitis, and the remainder were considered probable[18]. Studies investigating specific causative-agent meningitis have shown different patterns of agent-specific meningitis in various countries. In one study in Niger[19], 57.7% of cases were caused by Neisseria meningitidis. In another study in Mexico, more than 50% of cases were confirmed as Hib[20]. An in-depth description of epidemic meningitis should have detailed information on the causative agents, as different serotypes of meningococcal meningitis or other bacterial or viral agents are associated with different epidemic characteristics. Our study suffers from the fact that no information about the specific causative-agent meningitis was available. More in-depth studies are needed to explore more detailed and causative agent-specific characteristics of meningitis in the population.

## Conclusion

We described a broad picture of meningitis in Tehran, Iran. The age and sex distribution of cases mainly follow the same pattern seen in other countries and populations. We found a pattern of seasonality as well as small periodic incidence peaks.

## Competing interests

The authors declare that they have no competing interests.

## Authors' contributions

AMJ participated in design, conduct, and analysis of the study. ADE: participated in the design of the study. FA participated in conduct and analysis of the study.

MAH participated in analysis. YMJ participated in design, conduct and analysis.

MMG participated in the design of the study. All authors read and approved the final manuscript.
